# Prevalence and clinical picture of premenstrual syndrome in females from Bulgaria

**DOI:** 10.1186/s12991-019-0255-1

**Published:** 2020-01-15

**Authors:** Petranka Chumpalova, Rossitza Iakimova, Maya Stoimenova-Popova, Daniil Aptalidis, Milena Pandova, Maria Stoyanova, Konstantinos N. Fountoulakis

**Affiliations:** 1 University Multiprofile Hospital for Active Treatment “Doctor Georgi Stranski”, 8A Georgi Kochev Blvd., 5800 Pleven, Bulgaria; 2 Department of Psychiatry and Medical Psychology, Medical University—Pleven, 113, Storgozia Distr., 5800 Pleven, Bulgaria; 3 University Multiprofile Hospital for Active Treatment in Neurology and Psychiatry “Sveti Naum”, 1 Lyuben Russev Str., 1113 Sofia, Bulgaria; 4 Department of Psychiatry and Medical Psychology, Medical University—Sofia, 1 Georgi Sofiyski Blvd., 1000 Sofia, Bulgaria; 5 3rd Department of Psychiatry, Faculty of Medicine, School of Health Sciences, Aristotle University of Thessaloniki, Thessaloniki, Greece

**Keywords:** Premenstrual syndrome, Premenstrual dysphoric disorder, Prevalence, Clinical picture

## Abstract

**Background:**

Premenstrual syndrome (PMS) and its more severe form premenstrual dysphoric disorder (PMDD) are highly prevalent conditions, but there seems to be ethnic and cultural variances in their distribution.

**Aims:**

To explore the prevalence of PMS/PMDD and their typical clinical features in a Bulgarian population.

**Materials and methods:**

This investigation was designed and executed as a cross-sectional descriptive study. Three hundred and five conveniently recruited females with no psychiatric history filled in a self-evaluation questionnaire based on DSM-IV tapping on different symptoms of PMS. The prevalence of the conditions was calculated.

**Results:**

32.1% (*N *= 98) of the tested females (mean age 31.04 ± 6.31) suffered from PMS and 3.3% (*N *= 10) were diagnosed with PMDD. The leading symptoms in the sample were irritability, fatigue and changes in appetite, depressed mood, mood swings, and anxiety, and abdominal bloating, breast tension and tenderness. Most of the symptoms were moderately severe. Mild and moderate cases of PMS were near equally distributed and more frequent than severe ones.

**Conclusion:**

PMS is a common condition which is usually mildly expressed, but severe cases are not an exception. The clinical picture is dominated by almost equally distributed psychological and somatic symptoms.

## Background

Premenstrual syndrome (PMS) is broadly defined as a cluster of emotional, physical, and behavioural symptoms that arise around the end of the luteal phase and dissipate with the beginning of menstruation or briefly thereafter [1]. According to different investigations on the prevalence of PMS, its frequency varies considerably depending on the methodology and assessment instruments used [1–9].

Altogether results show, that up to 90% of women of reproductive age experience several premenstrual symptoms varying from mild to severe; around 20–40% of them experience PMS, and 2–8% suffer from premenstrual dysphoric disorder (PMDD) [10]. At the same time, it seems that the prevalence of the syndrome varies among cultures and ethnic groups [11, 12], although such a difference is not always to be found as shown in a study of females of European, East Asian, and South Asian origin [13]. Country-specific studies on the prevalence are necessary for proper and more accurate evaluation of the prevalence of the syndrome [14].

Having in mind the above-mentioned considerations, together with the lack of structured investigations on the topic in the Bulgarian population, we decided to examine the prevalence of PMS/PMDD and its characteristics.

## Materials and methods

### Study sample

The current investigation was designed as a cross-sectional descriptive study. The study sample consisted of 350 females of Bulgarian origin between 18 and 50 years of age with regular menstrual cycles with a length between 21 and 35 days who were recruited in outpatient settings. Forty-five of them dropped out because of inaccurate completion of the questionnaire or unwillingness to report an existing psychiatric condition. The final group consisted of 305 females. The participants were recruited randomly by visiting different companies, administrative offices, universities. Those women who agreed to participate and certified this by signing an informed consent form were interviewed about their body weight and gynaecological condition, including recent or present pregnancy, regularity and duration of menstrual cycle, use of contraceptives or other hormonal preparations. Co-morbid mental disorders were excluded by Mini International Neuropsychiatric Interview (M.I.N.I.6.0.).

Criteria for exclusion were lactation within 3 months prior to study, pregnancy, oral contraceptives use, co-morbid mental disorder, and use of psychopharmacological medicines for any reason.

PMS/PMDD were diagnosed by the criteria of the Diagnostic and Statistical Manual of Mental Disorders, fourth edition (DSM-IV) [15] and the American College of Obstetrics and Gynaecology (ACOG) [16]. We used a questionnaire based on the PSST (Premenstrual Screening Tool, Additional file 1) which is a self-evaluation instrument for a retrospective assessment of symptoms persisting for 2 weeks before menstruation in the preceding 12 months [17]. It assesses premenstrual symptoms, such as mood, anxiety, sleep, appetite, and somatic symptoms—breast tenderness, headaches, joint/muscle pain, abdominal bloating, weight gain, palpitations, hot and cold flashes. For the purpose of precise description of the clinical picture, we assessed this latter symptom group separately. The participants evaluate each symptom and the level of functional impairment (if present) on a 4-point Likert scale as “not at all”, “mild”, “moderate”, and “severe”. The following diagnostic criteria were used: mild/moderate PMS: 1. At least one of 1, 2, 3 4 is mild/moderate; 2. In addition at least four of 1–19 are mild/moderate; 3.20 is mild/moderate; PMDD: 1. At least one of 1, 2, 3, 4 is severe; 2. In addition at least four of 1–19 are severe; 3.20 is severe.

The investigation was approved by Ethics Committee Medical Center “Sveti Naum”. All participants signed an informed consent before initiating the study procedures.

### Statistical analyses

The data were analysed with the Statistical Package for Social Sciences version 13 (SPSS 13), whereby descriptive statistics and frequency analyses were followed by t-test. The *p*-level below 0.05 was considered as the criterion for statistical significance.

## Results

Ninety-eight females (32.1%) (mean age 31.04 ± 6.31) met the criteria for PMS and 207 (67.9%) (mean age 30.22 ± 5.37) did not. The two groups did not differ significantly by age (t (303) = 1.174, *p* = 0.241).

According to our data, psychological and somatic symptoms were almost equally represented in the PMS group. The most prevalent symptoms within the psychological type were irritability, fatigue and changes in appetite, depressed mood, mood swings, and anxiety, whereas among the most common somatic ones were abdominal bloating, breast tenderness, headache, and weight gain (Table 1).

**Table 1:** Prevalence of PMS symptoms in females with (*N* = 98) and without (*N* = 207) PMS

Symptoms	PMS	No PMS	Symptoms	PMS	No PMS
Psychological	*n *(%)	*n* (%)	Somatic	*n* (%)	*n* (%)
Depressed mood	77 (78.6)	54 (26.1)	Abdominal bloating	83 (84.7)	154 (74.4)
Irritability	86 (87.8)	103 (49.8)	Weight gain	48 (49.0)	73 (35.3)
Mood swings	75 (76.5)	53 (25.6)	Breast tension	80 (81.6)	168 (81.2)
Anxiety	69 (70.4)	19 (9.2)	Joint pain	29 (29.6)	34 (16.4)
Hopelessness	35 (35.7)	7 (3.4)	Muscle pain	28 (28.6)	29 (14.0)
Apathy	30 (30.6)	10 (4.8)	Headaches	53 (54.1)	85 (41.1)
Poor concentration	59 (60.2)	61 (29.5)	Palpitations	14 (14.3)	17 (8.2)
Fatigue	78 (79.6)	90 (43.5)	Hot and cold flashes	43 (43.9)	30 (14.5)
Changes in appetite	78 (79.6)	121 (58.5)			
Sweets craving	68 (69.4)	99 (47.8)			
Sleep changes	42 (42.9)	19 (9.2)			

*PMS* premenstrual syndrome; *N* sample size; *n* number of subjects experiencing the symptom

The better part of the symptoms were moderately severe (Tables 2, 3)

**Table 2:** Severity of psychological symptoms in females with PMS (*N* = 98)

	Severity	*n* (%)		Severity	*n* (%)
Depressed mood	Mild	37 (48.0)	Poor concentration	Mild	28 (47.5)
	Moderate	27 (35.1)		Moderate	21 (35.6)
	Severe	13 (16.9)		Severe	10 (16.9)
	*N*	77 (100)		N	59 (100)
Irritability	Mild	28 (32.6)	Fatigue	Mild	26 (33.3)
	Moderate	32 (37.2)		moderate	30 (38.5)
	Severe	26 (30.2)		Severe	22 (28.2)
	*N*	86 (100)		*N*	78 (100)
Mood swings	Mild	20 (26.7)	Changes in appetite	Mild	10 (12.8)
	Moderate	35 (46.6)		moderate	41 (52.6)
	Severe	20 (26.7)		Severe	27 (34.6)
	*N*	75 (100)		*N*	78 (100)
Anxiety	Mild	33 (47.8)	Sweets craving	Mild	11 (16.2)
	Moderate	26 (37.7)		moderate	35 (51.5)
	Severe	10 (14.5)		Severe	22 (32.3)
	*N*	69 (100)		*N*	68 (100)
Hopelessness	Mild	12 (34.3)	Sleep changes	Mild	13 (31.0)
	Moderate	13 (37.1)		moderate	19 (45.2)
	Severe	10 (28.6)		Severe	10 (23.8)
	*N*	35 (100)		*N*	42 (100)
Apathy	Mild	17 (56.7)			
	Moderate	9 (30.0)			
	Severe	4 (13.3)			
	*N*	30 (100)			

*PMS* premenstrual syndrome, *n* number of subjects experiencing the corresponding symptom severity, *N* number of subjects experiencing the symptom

**Table 3:** Severity of somatic symptoms in females with PMS (*N*  = 98)

	Severity	*n* (%)		Severity	*n* (%)
Abdominal bloating	Mild	17 (20.5)	Muscle pain	Mild	14 (50.0)
	Moderate	42 (50.6)		Moderate	14 (50.0)
	Severe	24 (28.9)		Severe	0 (0.0)
	*N*	83 (100)		N	28 (100)
Weight gain	Mild	26 (54.2)	Headaches	Mild	17 (32.1)
	Moderate	19 (39.6)		Moderate	22 (41.5)
	Severe	3 (6.2)		Severe	14 (26.4)
	*N*	48 (100)		N	53 (100)
Breast tension	Mild	17 (21.25)	Palpitations	Mild	6 (42.9)
	Moderate	38 (47.5)		Moderate	5 (35.7)
	Severe	25 (31.25)		Severe	3 (21.4)
	*N*	80 (100)		N	14 (100)
Joint pain	Mild	9 (31.0)	Hot and cold flashes	Mild	23 (53.5)
	Moderate	13 (44.8)		Moderate	16 (37.2)
	Severe	7 (24.1)		Severe	4 (9.3)
	*N*	29 (100)		N	43 (100)

*N* number of subjects experiencing the symptom; *n* number of subjects experiencing the corresponding symptom severity

**Fig. 1:**
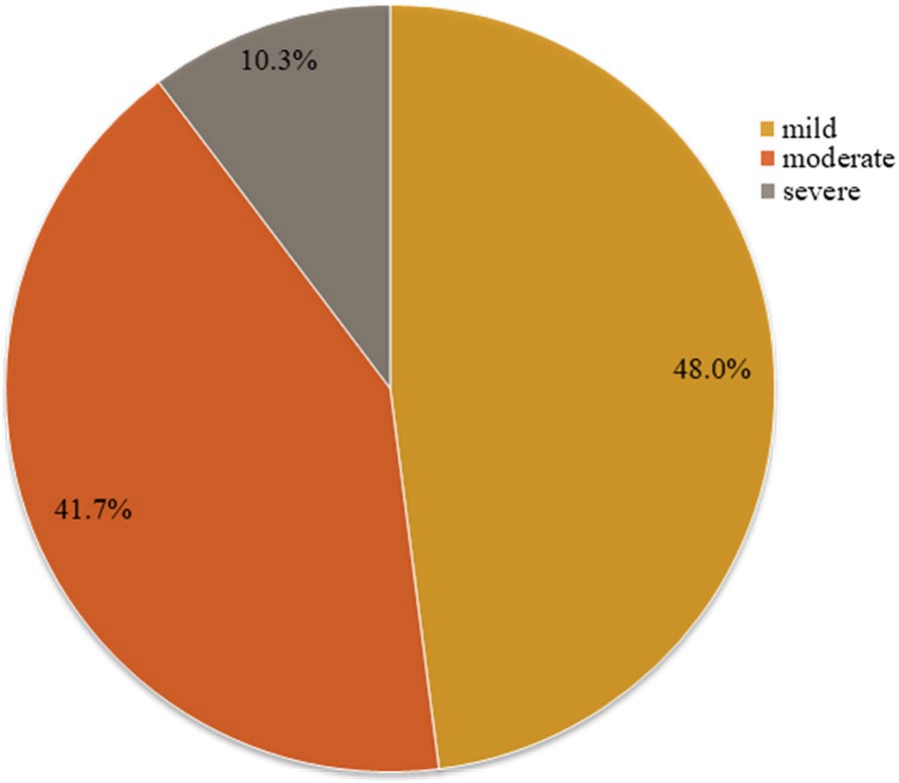
Severity of PMS (*N* = 98)

Of the tested subjects 15.4% (*N*  = 47) suffered from mild PMS and 13.4% (*N* = 41) from moderately severe. Severe syndrome corresponding to PMDD was registered in 3.3% (*N*  = 10) of the participants (Fig. 1).

67.9% of our sample or 207 women did not suffer PMS. 6.3% of them (13 women) did not experience any premenstrual symptoms. The remaining 93.7% (194 women) suffered sub-threshold symptoms usually denoted as “normal” premenstrual symptoms. Most commonly reported were irritability (49.7%), increased appetite (58.5%), fatigue (43.5%), breast tension (81.1%), abdominal bloating (74.4%), increased weight (35.3%) (Tables 4, 5).

**Table 4:** Severity of psychological symptoms in females without PMS (*N*  = 207)

Symptom	Severity	No PMS—n (%)	Symptom	Severity	No PMS—n (%)
Depressed mood	Mild	36 (66.7)	Poor concentration	Mild	36 (59.0)
	Moderate	14 (25.9)		Moderate	19 (31.2)
	Severe	4 (7.4)		Severe	6 (9.8)
	*N*	54 (100)		*N*	61 (100)
Irritability	Mild	42 (40.8)	Fatigue	Mild	54 (60.0)
	Moderate	51 (49.5)		moderate	23 (25.6)
	Severe	10 (9.7)		Severe	13 (14.4)
	*N*	103 (100)		*N*	90 (100)
Mood swings	Mild	41 (77.4)	Changes in appetite	Mild	54 (44.6)
	Moderate	12 (22.6)		moderate	31 (25.6)
	Severe	0 (0.0)		Severe	36 (29.8)
	*N*	53 (100)		*N*	121 (100)
Anxiety	Mild	15 (78.9)	Sweets craving	Mild	39 (39.4)
	Moderate	4 (21.1)		moderate	33 (33.3)
	Severe	0 (0.0)		Severe	27 (27.3)
	*N*	19 (100)		*N*	99 (100)
Hopelessness	Mild	5 (71.4)	Sleep changes	Mild	8 (42.1)
	Moderate	2 (28.6)		moderate	11 (57.9)
	Severe	0 (0.0)		Severe	0 (0.0)
	*N*	7 (100)		*N*	19 (100)
Apathy	Mild	10 (100)			
	Moderate	0 (0.0)			
	Severe	0 (0.0)			
	*N*	10 (100)			

*PMS*: premenstrual syndrome; *n*: number of subjects experiencing the corresponding symptom severity; *N*: number of subjects experiencing the symptom

**Table 5:** Severity of somatic symptoms in females without PMS (N = 207)

Symptom	Severity	No PMS—* n* (%)	Symptom	Severity	No PMS—* n* (%)
Abdominal bloating	Mild	82 (53.2)	Muscle pain	Mild	15 (51.7)
	Moderate	40 (26.0)		Moderate	12 (41.4)
	Severe	32 (20.8)		Severe	2 (6.9)
	*N*	154 (100)		*N*	29 (100)
Weight gain	Mild	50 (68.5)	Headaches	Mild	38 (44.7)
	Moderate	21 (28.8)		Moderate	26 (30.6)
	Severe	2 (2.7)		Severe	21 (24.7)
	*N*	73 (100)		*N*	85 (100)
Breast tension	Mild	70 (41.7)	Palpitations	Mild	11 (64.7)
	Moderate	70 (41.7)		Moderate	5 (29.4)
	Severe	28 (16.6)		Severe	1 (5.9)
	*N*	168 (100)		*N*	17 (100)
Joint pain	Mild	14 (41.2)	Hot and cold flashes	Mild	13 (43.3)
	Moderate	12 (35.3)		Moderate	13 (43.3)
	Severe	8 (23.5)		Severe	4 (13.4)
	*N*	34 (100)		*N*	30 (100)

*N* number of subjects experiencing the symptom, *n* number of subjects experiencing the corresponding symptom severity

## Discussion

Our results replicate relatively well what is known from prior research in the field [5, 8, 9, 18]. Despite the use of different diagnostic instruments, the prevalence of PMS usually varies around 20–40% [10]. This is entirely comparable to our data, namely 32.1%. Our results on the prevalence of PMDD—3.3%, are also similar to previous reports in the literature—3–8% [8, 9, 12, 19, 20], although much higher rates have also been reported [6].

The estimates of the prevalence of PMS differ also among cultures and ethnic groups. A study among Japanese women reports low levels of both PMS and PMDD—5.3% and 1.2%, respectively. The authors assume that this is a consequence of the traditional Confucian ethics, which subdue individual welfare to the group wellbeing and as a result women have difficulties verbalizing their complaints [21]. On the contrary, two consecutive studies in the Pakistani population find higher prevalence of PMS—92.4% and 98.2%, respectively [11, 12]. The authors explain it partly with ethnic specificities. But this data is not confirmed by a Canadian team of investigators who targeted 4 ethnic groups—Caucasian, East Asian, South Asian, and a fourth group, including other ethnicities. They do not find any significant differences among groups which the authors relate to the unification of lifestyle and health-related attitudes in modern society [13]. Regarding the Balkans, the available data do not prove to be considerably different either from the data for Europe or from ours—the prevalence of PMS among Greek students is 25.7% [22] and in Turkey—16% [23].

As mentioned above, differences in the diagnostic instruments used also play a role in the estimates of the prevalence of PMS. For example, a Saudi Arabian team of researchers used a questionnaire based on the definition of the American College of Obstetrics and Gynecology and found PMS in 35.6% of the sample, from which 22.4% severe [24]. When DSM-IV is used, the prevalence of PMS varies from 1.2% in a Japanese community sample to 17.9% among Brazilian students [21, 25], 29% in Ukraine [5], and 37.3% in Myanmar [6]. We also use the DSM-IV definition and our results are comparable to those from Ukraine. This could probably be explained with cultural similarities.

Furthermore, our data prove similar to the results of three other studies that like us, used the PSST questionnaire—an Israeli team that reports 25.6% prevalence for PMS and 9.9% for PMDD [9], an Indian group that finds PMS in 18.4% of its sample and PMDD in 3.4%, resp. [20], and Iranian researchers that observe PMS in 30.7% and PMDD in 12.9% [8]. Comparable results were obtained in two other studies—one in Turkey—16% [23], and another one in Uzbekistan—28.1% [7], that used the Premenstrual Symptoms Form (PAF). These results are also close to ours—32.1%.

Our findings on the nearly equal distribution of both symptom types in the clinical picture of PMS are also in accord with the data from other researchers [8], although there are also reports with different results [26]. All of the following have been reported as core symptoms of the syndrome: anxiety, fatigue, depression and tension, headaches, skin disturbances, cramps, breast aches/tension, weight gain and abdominal/extremities bloating, anger, irritability, mood changes, changes in appetite and sleep pattern, specific foods craving, reduced interest in activities [19, 27]. Most commonly described as severely disabling are irritability and tension, and as causing most severe distress—headaches [28].

The results from our sample are all in all congruent to these findings with irritability being practically the most prevalent psychological symptom and the third most commonly severely expressed after changes in appetite and sweets craving, and headaches being the third in row of prevalence as well as severity among somatic symptoms. In addition, we identified breast tension and tenderness and abdominal bloating as core somatic symptoms.

## Limitations

The presented study has certain limitations. The sample size is small and needs to be enlarged in order to obtain representative results. The data on the gynaecological condition of women and the characteristics of their menstrual cycle are only anamnestic. The patients were not prospectively followed up.

## Conclusion

For the first time, our study estimates the prevalence rate and describes the typical clinical signs of PMS/PMDD among Bulgarian women. PMS is broadly distributed and occurs at a similar rate in Bulgaria as in other European countries. It is most commonly mildly expressed and severe cases are rare. The clinical picture consists of nearly evenly distributed psychological and somatic symptoms of which most common are irritability, changes in appetite, breast tension and tenderness, abdominal bloating.

## Supplementary information

**Additional file 1.** Premenstrual Symptom Screening Tool.

## Data Availability

The datasets used and/or analysed during the current study are available from the corresponding author on reasonable request.

## Abbreviations

ACOG: American College of Obstetrics and Gynaecology
DSM-IV: Diagnostic and Statistical Manual of Mental Disorders
PAF: premenstrual symptoms form
PMDD: premenstrual dysphoric disorder
PMS: premenstrual syndrome
PSST: premenstrual screening tool
SPSS: statistical package for social sciences
