# Supplementary material for: Prevalence and clinical picture of premenstrual syndrome in females from Bulgaria
Source: Ann Gen Psychiatry. 2020 Jan 15;19:3. doi: 10.1186/s12991-019-0255-1 (PMC6964059; doi:10.1186/s12991-019-0255-1)
Supplement: Supplementary file 1 — Additional file 1. Premenstrual Symptom Screening Tool. [file 12991_2019_255_MOESM1_ESM.docx]

Additional file 1

Premenstrual Symptom Screening Tool

(please, mark an “X” in the appropriate box)

Do you experience some or any of the following premenstrual symptoms which start before your period and stop within a few days of bleeding?

| Symptom | Not at all | Mild | Moderate | Severe |
| --- | --- | --- | --- | --- |
| 1. Do you get sad, depressed, or tearful? |  |  |  |  |
| 2. Do you get irritable, angry, or nervous? |  |  |  |  |
| 3. Does your mood change easily and for no substantial reason? |  |  |  |  |
| 4. Do you feel anxious, tense, or restless? Do you feel like losing control over yourself? |  |  |  |  |
| 5. Do you feel useless, inefficient, hopeless about your future, guilty? |  |  |  |  |
| 6. Do you feel less interested in important things for you? |  |  |  |  |
| 7. Do you feel more distracted; do you notice that it is harder for you to concentrate? |  |  |  |  |
| 8. Do you get more easily fatigued, do you feel your energy level (level of activity) lowered? |  |  |  |  |
| 9. Does your appetite change - increase or decrease? |  |  |  |  |
| 10. Do you feel increased craving for sweet foods? |  |  |  |  |
| 11. Do you have trouble sleeping - falling asleep, midnight awakenings, early morning awakening? |  |  |  |  |
| 12. Does your stomach bloat? |  |  |  |  |
| 13. Does your weight increase? |  |  |  |  |
| 14. Do you feel your breast tense and/or do you get unpleasant (painful) sensation by touch? |  |  |  |  |
| 15. Do you experience joint aches? |  |  |  |  |
| 16. Do you experience muscle aches? |  |  |  |  |
| 17. Do you have headaches? |  |  |  |  |
| 18. Do you have palpitations? |  |  |  |  |
| 19. Do you feel shivering, shaking, do you have hot and cold flashes? |  |  |  |  |

If you have answered “YES” to 5 or more of the above questions, please answer the following:

|  | Not at all | Mild | Moderate | Severe |
| --- | --- | --- | --- | --- |
| Do the symptoms listed above interfere with your daily life; do they worsen your ability to work; do they interfere with your relationships with your family, colleagues, friends? |  |  |  |  |

**Scoring**

The following criteria must be present for a diagnosis of PMDD

1. At least one of 1, 2, 3, 4 is severe;
2. In addition at least four of 1 to 19 are severe;
3. 20 is severe.

The following criteria must be present for a diagnosis of mild to moderate PMS

1. At least one of 1, 2, 3 4 is mild to moderate;
2. In addition at least four of 1 to 19 are mild to moderate;
3. 20 is mild to moderate.
